# Research on Multi-Agent Semantic Communication Framework Based on Comparative Learning Joint Optimization

**DOI:** 10.3390/s26102963

**Published:** 2026-05-08

**Authors:** Hong Yang, Hongyan Li, Honggang Chen, Lijuan Wang, Ji Li, Linbo Qing, Xiaohai He

**Affiliations:** 1 College of Electronics and Information Engineering, Sichuan University, Chengdu 610065, China; yhscu@scu.edu.cn (H.Y.); honggang_chen@scu.edu.cn (H.C.); wanglijuan@stu.scu.edu.cn (L.W.); liji@stu.scu.edu.cn (J.L.); qing_lb@scu.edu.cn (L.Q.); hxh@scu.edu.cn (X.H.); 2 Department of Industry and Information Technology of Xizang Autonomous Region, Lhasa 850014, China

**Keywords:** semantic communication, multi-agent, joint optimization, comparative learning

## Abstract

With the rapid development of intelligent services, communication objectives are shifting from humans to multi-agent (MA) systems. This transition necessitates new communication paradigms capable of supporting real-time perception, decision-making, and collaboration among agents. Semantic communication (SeC) focuses on the efficient transmission and accurate understanding of information “meaning” and is well-suited to meet the needs of Mas, such as collaborative perception, reasoning, and decision-making. However, the transmission of semantic information is still constrained by dynamic environments and the diversity of MA tasks. To address these challenges, this work proposes a COmparative learning Joint Optimal (COJO) SeC framework. This work makes three main contributions: first, it jointly optimizes the image reconstruction and classification functions designed for multi-task semantic objectives under different channel conditions, thereby improving the overall task performance of the system; second, based on input image features, compression ratio, task requirements, and channel conditions, an enhanced further compressor is designed, which obtains a training-based mask to significantly reduce the volume of transmitted data; finally, to prevent the loss of key semantic information in multi-task scenarios under channel constraints, it designs a task-driven end-to-end semantic communication training scheme.

## 1. Introduction

With the rapid development of intelligent technology, Internet of Things (IoT) and 6G communication technology, communication entities are evolving from humans to multi-agent (MA) systems capable of environmental perception, semantic understanding, and autonomous decision-making [1]. It is anticipated that MA systems will be widely deployed in various fields [2], such as collaborative service robots, Unmanned Aerial Vehicle (UAV) swarms, and connected vehicles. MA communication aims to provide global connectivity, controllable information exchange and task collaboration for heterogeneous agents with different forms, capabilities, and users’ demands [3,4]. Compared with traditional mobile terminals and IoT devices, MA systems can actively initiate communication and collaborate to complete complex tasks assigned by humans. It is crucial that communication within MA systems is no longer constrained by human sensory or cognitive limitations. Instead, MA systems can directly exchange semantic-level information required to perform tasks, rather than transmitting raw text, images, or audiovisual data [5].

Semantic communication (SeC) has emerged as a revolutionary communication paradigm, abandoning the traditional “transmit first, compute later” model and adopting a “compute first, transmit later” approach [5,6,7,8,9]. Its core principle is to transmit only the semantic information relevant to the task, significantly reducing communication overhead and improving efficiency while ensuring task completion [5]. Compared with traditional bit-level transmission, SeC supports cross-modal information representation and robust end-to-end transmission [10]. It has been recognized by 3GPP as a key enabling technology for 6G [11] and has been proven to be particularly suitable for MA communication scenarios.

The core of MA systems lies in “understanding and decision-making”, which can be directly realized through the exchange of abstract semantic features. Recent studies in semantic communication have achieved remarkable progress in text, speech, and image-oriented SeC by leveraging convolution neural networks (CNNs), Transformer architectures, and generative models [12,13,14]. SeC exhibits advantages in both single tasks [15,16,17,18] and multiple-task applications [19,20,21,22,23,24,25,26,27,28]. Reference [18] tackled the conflict between “high bandwidth demand” and “low semantic fidelity” in conventional wireless image transmission and proposed a Transformer-based wireless image transmission model (WITT) that developed rapidly, as it is not restricted to fixed tasks and can support customized task configurations according to diverse requirements. Reference [19] solved the problem that “static encoding strategies are difficult to adapt to dynamic data and task requirements” in semantic communication, and put forward a data-adaptive semantic communication system. A generative semantic communication framework was introduced to satisfy the “dual-task collaboration” demand of image transmission and semantic segmentation [20]. To alleviate the challenges of “inter task resource competition” and “the no-applicability of traditional rate distortion theory to semantic-oriented optimization” in multi-task semantic communication, reference [21] proposed a multi-task semantic communication scheme based on the extended rate distortion theory. A Task Unaware Transmitter semantic communication system is proposed in reference [25], in order to address the issue of “the transmitting end needs to be aware of the receiving end’s tasks in advance, making it difficult to adapt to dynamic task changes” in semantic communication.

In recent years, generative semantic communication has gradually emerged as a novel research direction. It is highly applicable to scenarios that center on “semantic priority, no need to restore the original carrier”, such as low bit rate image/video transmission, meta universe multimodal interaction, semantic decision-making in edge computing, IoT sensor data semantic transmission, etc., which can achieve high-quality content generation at very low bit rates, while adapting to cross-modal semantic transmission requirements (such as image generation text, text generation and image transmission) [26]. Accordingly, for scenarios requiring the accurate restoration of original information, conventional reconstruction-based semantic communication is preferred, with the core optimization objective centered on semantic fidelity. In contrast, for application demands characterized by low-bit-rate, strong anti-interference capability and high-quality semantic regeneration, generative semantic communication should be prioritized, which focuses on generative model adaptation and semantic consistency constraint.

However, multi-agent (MA) communication faces unique requirements and inherent challenges, and dedicated research in this field remains insufficient. A critical unresolved issue lies in the high mobility and multi-task operation of MA systems, which generally execute missions in complex environments accompanied by frequent wireless channel fluctuations. Such conditions mandate the communication system to possess sufficient flexibility and robustness, so as to adapt semantic representation to dynamically changing task demands and time-varying communication environments. Moreover, transmission strategies and semantic transmission rates need to be adaptive, adjusted according to real-time channel conditions. To tackle the above challenges, this work focuses on multi-task MA application scenarios. Leveraging contrastive learning, the proposed framework jointly optimizes image reconstruction quality and classification accuracy, thereby enhancing the multi-task processing capability and overall transmission performance of semantic communication systems. The main contributions of this paper are summarized as follows:

(1) To enhance task efficiency and operational stability in multi-task, multi-agent (MA) environments, this paper proposes a COntrastive learning Joint Optimal (COJO) semantic communication (SeC) scheme, which integrates image reconstruction and classification functions to satisfy multi-task semantic objectives. The designed Task Recovery Module (TRM) is developed to balance reconstruction distortion and semantic distortion, enabling high-quality image reconstruction and accurate MA image processing tasks (e.g., classification) under diversified channel conditions. In particular, when channel quality deteriorates, the proposed scheme can dynamically determine task priorities according to practical MA task requirements and further adapt the transmission strategy correspondingly.
(2) To reduce the semantic transmission bit rate for MA systems, a Task Compression Module (TCM) is designed to perform further semantic compression. This module is developed based on input image features, compression ratios, task requirements, and channel conditions, and a mask is obtained through training. The TCM dynamically selects different masks according to actual scenarios, which significantly reduces the volume of transmitted data while ensuring the stability of MA task performance.
(3) Under channel constraints, an end-to-end training scheme driven by both image reconstruction and classification objectives is designed to avoid key semantic loss in multi-task scenarios. Considering the inherent feature space mismatch between the semantic information required for image classification and reconstruction tasks, a weighted joint loss function (JLF) is developed to realize the collaborative optimization of the two tasks. The total loss consists of weighted classification cross-entropy loss and reconstruction fidelity loss. By balancing the optimization gradients of the two tasks, the proposed scheme alleviates the feature objective conflict in MA systems.

The remainder of this work is structured as follows: Section 2 elaborates on the framework of the proposed COJO scheme; Section 3 presents the simulation results of the proposed method along with corresponding analysis; and Section 4 summarizes the conclusion of this work.

## 2. The Proposed COJO Scheme

**Figure 1:**
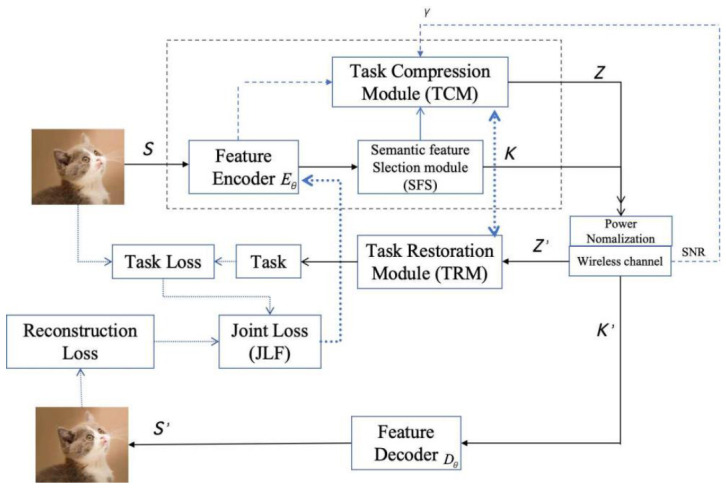
The detail of COJO scheme. The different arrows mean different signal transmission on it.

An overview of the COJO architecture for multi-tasking applications is given in Figure 1, which includes the feature encoder $E_{\theta }$, Feature Decoder $D_{\varphi }$, joint loss function (JLF), Semantic Feature Selection (SFS) module, Task Compression Module (TCM) and Task Restoration Module (TRM).

Inspired by the recent advance of SeC [13], this work represents the processed image $d_{in}$ dimension as $S\in {\mathbb{R}}^{d_{in}}$. Then *S* is encoded by the Wireless Image Transmission Transformer (WITT) Encoder $E_{\theta }$ to get the semantic feature information *K*, which is regarded as the first stage of compression:

(1) $$K=E_{\theta }(S)$$

Based on the findings in [29], the image classification task focuses on extracting high-level abstract semantic features, emphasizing inter-class differences and semantic in-variance while being insensitive to local redundant details. In contrast, the image reconstruction task concentrates on capturing low-level spatial structures, textures and other high-frequency information, prioritizing pixel-level fidelity without requiring semantic features. Obvious feature space mismatch exists between the two tasks. Accordingly, inspired by [29], we design a TCM to further compress semantic features. The TCM adaptive selects task-specific compressed features Z for different task demands:

*Z* = T(*K*) (2)

The simultaneous transmission of the generalized semantic feature *K* and task feature *Z* over wireless channels is inevitably affected by channel noise. To endow the system with the capability of autonomously selecting task-critical semantic features for diverse requirements, as well as reducing data redundancy, improving compression performance and enhancing communication efficiency, power normalization is performed on K and Z prior to transmission to meet the average power constraint. The transmission procedure of *Z* is described as follows, with the AWGN channel taken as an example:

(3) Y=Z+υ

where υ represents the channel noise, $\upsilon \in CN(0,\omega^{2}I)$ represents additive Gaussian white noise, CN represents complex Gaussian distribution function, and $\omega^{2}$ represents noise power.

Then, the processed features are directly transmitted over the wireless channel and received by the receiver. The reconstructed image S′ is decoded via the WITT module and then fed into the TRM for final task result acquisition.

Overall, the proposed scheme can preserve critical features in the image semantic encoder and selectively transmit them. After transmission over a noisy channel, the receiver acquires the key information required for image reconstruction and further executes the classification task smoothly. This process fully reflects the compression capability and robustness of the model.

### 2.1. The Joint Loss Function (JLF) Method

Since this work develops an image-oriented model for joint tasks, the designed JLF is capable of jointly considering both image reconstruction and classification objectives. During the training phase, the JLF comprehensively accounts for errors derived from reconstruction and classification, so as to adaptively balance dual tasks or prioritize a single task as needed. Meanwhile, channel conditions and task demands are also incorporated into the JLF to guide the optimization of model parameters throughout the training process.

This article uses the most commonly used Mean Squared Error (MSE) loss function as the reconstruct loss function $\xi_{rec}$:

(4) $$\zeta_{rec}=E_{\left(s,s^{\prime }\right)}\left\{\frac{1}{n}||s-s^{\prime }|{|}_{2}^{2}\right\}$$

In image task semantic communication models, contrastive learning [22] is often used as a classification loss function $\xi_{cl}$:

(5) $$\xi_{cl}=\frac{1}{2N}\sum_{i=1}^{N}\left(y_{i}\cdot D^{2}+\left(1-y_{i}\right)\cdot \max{\left(0,m-D\right)}^{2}\right)$$

where $y_{i}\in \left\{0,1\right\}$ represents the label. If it is a positive sample pair, then $y_{i}=1$; if it is a negative sample pair, then $y_{i}=0$. The Euclidean distance between the sample pairs m is a constant and called the “distance boundary”, which represents the minimum distance between negative sample pairs, and is commonly used to control the separation of negative samples. N is the total number of sample pairs.

The optimization objective of positive sample pairs is to minimize the distance between $s_{1}$ and $s_{2}$. On the other side, for the negative sample pairs, the optimization objective of negative sample pairs is to make the distance between the sum greater than a certain boundary, even if the distance between negative samples is as large as possible. Thus, this work designs a weighted joint loss function (JLF), which is the simplified version of reference [29], to achieve the collaborative optimization of classification and reconstruction. The total loss is composed of weighted classification cross entropy loss and reconstruction fidelity loss. The entire model adopts an end-to-end training approach to ensure task integrity. From the perspective of joint optimization, the JLF contributes significantly, as it is built on commonly used reconstruction loss functions $\xi_{rec}$ and classification loss functions $\xi_{cl}$, and set λ = [0, 1] as a weight factor to balance the losses of these two parts. The joint semantic loss function $\xi_{jo\mathrm{int}}$ of the algorithm proposed in this work can be expressed as:

(6) $$\xi_{jo\mathrm{int}}=\lambda \xi_{rec}+\left(1-\lambda \right)\xi_{cl}$$

The joint loss function aims to minimize the overall loss, thereby achieving a favorable trade-off between image reconstruction and classification performance. Weight coefficients are introduced to balance the optimization gradients of the two tasks, and a task-aware mechanism is adopted to mitigate conflicts between their feature learning objectives. Accordingly, the minimization objective of the proposed JOSRC framework can be formulated as follows:

(7) $$\underset{\theta }{\min}\xi_{jo\mathrm{int}}=\underset{\theta }{\min}\left(\lambda \xi_{rec}+\left(1-\lambda \right)\xi_{cl}\right)$$

where *θ* is the parameter of the COJO scheme.

To ensure task integrity, this structure adopts an end-to-end training approach, which enables the preservation of critical features in the image reconstruction encoder followed by selective transmission. After transmission over wireless channels, the receiver acquires the key information required for image reconstruction and then smoothly executes the classification task, thereby verifying the compressibility and robustness of the model.

### 2.2. The Designed Task Compression Module (TCM)

**Figure 2:**
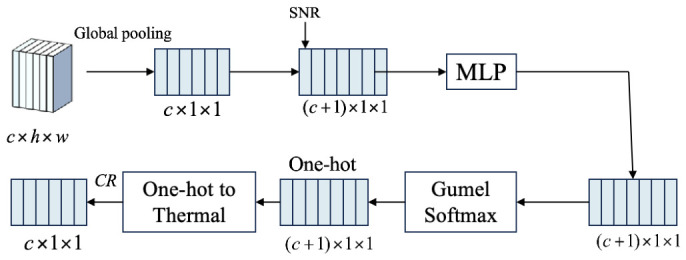
The TCM structure.

The TCM conducts further compression after the feature encoder and generates an MA system according to input image features (K), compression ratio (R), and signal-to-noise ratio (SNR). Combined with image characteristics and real-time channel SNR, the TCM adaptively selects the optimal compression ratio R for experimental deployment. The detailed structure is illustrated in Figure 2.

Specifically, the TCM first concatenates the mean value of input feature K with the SNR. Through several fully connected layers, five weight vectors corresponding to different compression strategies are generated. Subsequently, the Gumbel Softmax method is adopted to sample an initial MA system from the five vectors. By calculating the residual between the sampled MA system and a deviation term and performing residual optimization, the final MA system is obtained. Ultimately, the One hot to thermo function converts the processed MA system into a heat map form and outputs the optimized MA system.

### 2.3. The Designed Task Restoration Module (TRM)

**Figure 3:**
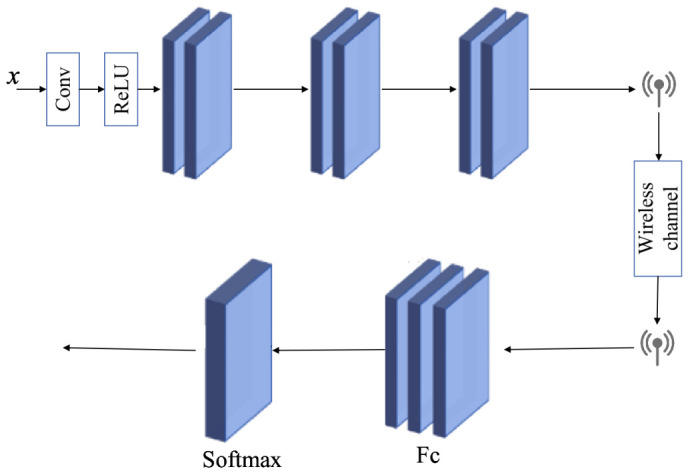
The TRM structure.

The TRM adopts a three residuals block (Res-block) structure, combined with a convolution layer, as shown in Figure 3. Due to further compression, the ResNet32 network was chosen to accelerate the convergence speed of the model, with output dimensions set to [16, 32, 64]. Each Res-block contains multiple convolution layers with skip connections between the input and output, allowing information to be directly transmitted in the network. This not only avoids signal attenuation in deep networks, but also facilitates gradient flow.

## 3. Experimental Results

This section is organized with subheadings to provide a concise and precise description of the experimental results, along with their in-depth interpretation and the key conclusions derived therefrom. It focuses on presenting the experimental findings objectively, analyzing the underlying mechanisms that led to such results, and summarizing the core insights and implications of the experiments for the proposed scheme.

### 3.1. Experimental Setup

**Datasets:** The proposed COJO scheme is trained and evaluated on image datasets. Specifically, the CIFAR-10 dataset is employed for both training and testing processes. Subsequently, generalization experiments of the proposed algorithm are conducted using the CIFAR-100, Kodak, and STL-10 datasets to verify its adaptability across different data distributions.

**Scheme Description:** The proposed COJO scheme is compared with several typical semantic communication (SeC) frameworks, including the SemCC model [22] and DeepSC model [25]. Both COJO and SemCC [22] are optimized for the joint task of image reconstruction and classification, and adopt contrastive learning to construct the classification loss function. In addition, the encoder–decoder architecture adopted by DeepSC [25] shares a similar structural design with the COJO scheme.

In the experiments, the compression ratio is set within the range of [1/24, 1/2.5]. All the training and testing experiments are implemented over AWGN channels to simulate image transmission and reconstruction under diverse compression ratios. To achieve a fair comparison of reconstruction and classification performance among the three models, all schemes are trained and tested at fixed SNR of 5 dB and 20 dB. Furthermore, to validate the channel adaptability of the proposed algorithm, additional evaluations are carried out under Rayleigh fading channels.

**Training Details:** First, we load the pre-trained weights of the classification network and conduct end-to-end joint training with the semantic reconstruction codec. Meanwhile, data augmentation is applied to the training images. In the training stage, image data is shared between the transmitter and receiver, and the receiver can access original images and corresponding labels. The semantic reconstruction loss and image classification loss are calculated individually and fused to construct the joint loss. In accordance with Equation (7), the joint optimization loss is minimized. Finally, the model is iteratively optimized to lower the overall loss and attain satisfactory comprehensive performance, as shown in the Algorithm 1.
**Algorithm 1.** Training steps for COJO in image multi-task**Input**: images “S”, real label “Labels”, Batch-size is “B”, Learning rate is “η”;**Output**: Semantic Encoder, $E_{\theta },$ Semantic Decoder $D_{\varphi },$ Classifier $T_{\Omega };$1:  Setting the training round counter i=1;2: When the total number of training rounds has not been reached, perform the following steps:3:     Randomly select samples from the training data set $S_{i};$4:     Semantic encoding of samples S to obtain $K_{i}=E_{\theta }(S_{i});$5:     Randomly generate AWGN channel noise $\upsilon_{i}∼CN(0,\omega^{2}I)$ based on signal-to-noise ratio $\lambda_{i};$6:     Obtain semantic features after adding noise $Y_{i};$7:     The receiver decodes $Y_{i}$ and reconstructs the data $S_{i}^{\prime }=D_{\varphi }(Y_{i});$8:     The classifier $H_{i}={Τ}_{\Omega }(S_{i}^{\prime })$ classifies the reconstructed image $S_{i}^{\prime };$9:     Calculate the loss value $\xi_{jo\mathrm{int}}$ and update $E_{\theta },$
$D_{\varphi }$ and $T_{\Omega };$10:     i=i+1;11:  End of training.

**Evaluation Methods:** This work considers the joint optimization of image reconstruction and classification tasks; accordingly, a broad range of evaluation metrics are employed, including the pixel-level metric Peak Signal-to-Noise Ratio (PSNR) and the perceptual metric Average Accuracy (AAC), to assess the performance of the proposed framework. Such joint evaluation can provide a more comprehensive analysis and clear improvement directions for model optimization, while guiding the task balance under joint optimization scenarios. To verify the effectiveness of the proposed algorithm, testing experiments are conducted from seven aspects, specifically: (1) the performance of the TCM at low compression rates; (2) the performance of different COJO scheme variants; (3) the performance of COJO under different compression ratios (R); (4) a framework complexity experiment; (5) a subjective comparison of image reconstruction quality; (6) a channel adaptability experiment; (7) a generalization experiment.

### 3.2. Experiments Results and Analysis

#### 3.2.1. The Performance of TCMs for Lower Rates

**Figure 4:**
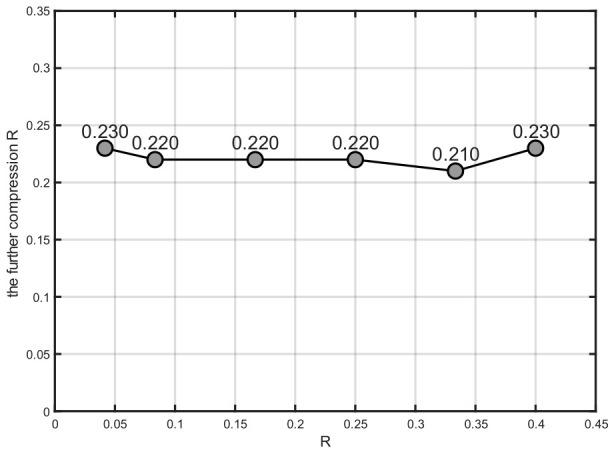
The further compression ratios of TCM at different compression ratios.

**Figure 5:**
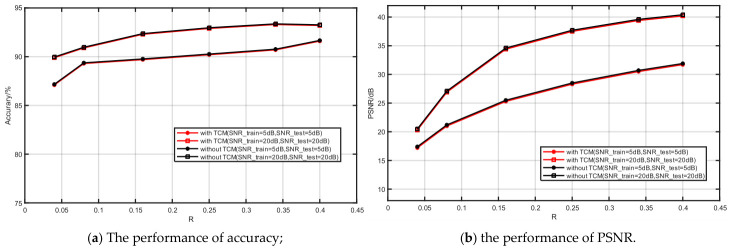
The impact of TCM.

To reduce the model compression ratio, the proposed COJO scheme compresses semantic features via semantic encoding, and further introduces the TCM for secondary compression. All experimental results in this work are obtained under further compression conditions, where the resultant bit rate is reduced to approximately 20% of the original, as illustrated in Figure 4. To verify the effect of the TCM on overall performance, ablation experiments with and without TCM are designed to compare image reconstruction quality and classification accuracy, as presented in Figure 5.

Figure 5a,b illustrate the impact of the TCM (with vs. without) on the performance of the COJO scheme in image reconstruction and classification tasks at SNRs of 5 dB and 20 dB, respectively. As shown in Figure 5a, further compression using the TCM has almost no impact on the model’s image classification accuracy, which maintains a stable trend without significant degradation or fluctuation. Specifically, the integration of the TCM does not exert a notable influence on the classification accuracy, indirectly verifying that the TCM achieves effective feature compression while ensuring classification performance.

From Figure 5b, it can be observed that the model’s reconstruction PSNR value exhibits a slight improvement (with a maximum increase of less than 0.2 dB) when the TCM is not used. This experimental result demonstrates that the TCM does not severely compromise the model’s reconstruction performance. Even without the TCM, the model’s reconstructed PSNR value still outperforms those of the comparison methods reported in the literature.

According to the experimental results in Figure 4 and Figure 5, the proposed COJO scheme can maintain and even boost model performance while achieving more efficient semantic compression. It effectively alleviates transmission bandwidth pressure, which demonstrates its superiority in joint image reconstruction and classification tasks for MA scenarios.

#### 3.2.2. The Performance of Different COJO Scheme Based on Joint Optimal

**Figure 6:**
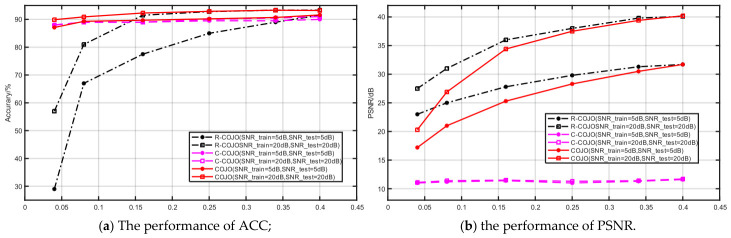
The performance of R-COJO, C-COJO and COJO.

This work focuses on the joint optimization of image reconstruction and classification tasks. Since the COJO framework adopts the proposed joint loss function (JLF), ablation experiments are designed based on this loss function to verify its effectiveness. For the fairness of comparison, only the loss function is adjusted during the experiment, and three model variants are established to compare their classification and reconstruction performance: the R-COJO scheme (considering only reconstruction loss), the C-COJO scheme (considering only classification loss), and the COJO scheme (adopting the joint loss function), as illustrated in Figure 6. The corresponding weight coefficients λ are set to 0, 0.5, and 1, respectively. All the experiments are trained and tested under fixed SNRs of 5 dB and 20 dB, with compression ratios R ranging from [1/24, 1/2.5].

Figure 6a presents the classification accuracy of the three models on the CIFAR-10 dataset, while Figure 6b shows their PSNR values for image reconstruction on the same dataset. As observed in Figure 6a, the classification accuracy of the COJO scheme is higher than that of the C-COJO scheme and significantly higher than that of the R-COJO scheme. From Figure 6b, it can be seen that at low compression rates, the reconstruction PSNR of COJO is lower than that of R-COJO but much higher than that of C-COJO. As the compression ratio R gradually increases, the gap in reconstruction PSNR between COJO and R-COJO narrows progressively, and eventually, COJO achieves performance consistent with or even superior to R-COJO. Overall, the COJO scheme can effectively balance reconstruction and classification performance, thereby achieving optimal comprehensive performance for multi-task MA applications.

#### 3.2.3. The Performance of COJO Based on Different Compression Ratio

**Figure 7:**
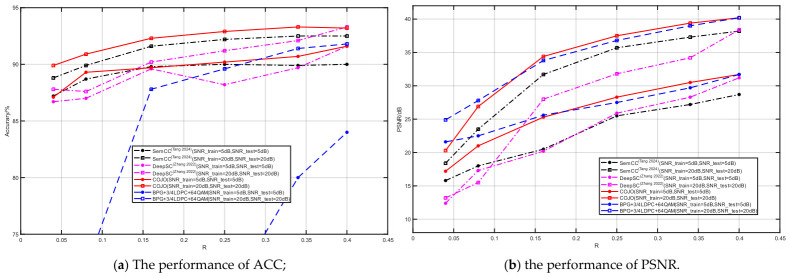
The performance of COJO based on different R [22,25].

The proposed COJO scheme and SemCC [22] are both optimized for the joint task of image reconstruction and classification, and both adopt contrastive learning as the classification loss function. They are also compared with DeepSC [25], as illustrated in Figure 7. To ensure a fair comparison of the reconstruction and classification performance among the three schemes, this experiment is conducted under fixed SNR conditions, with specific SNRs set to 5 dB and 20 dB. Notably, the encoder–decoder architecture of DeepSC [25] is modified to be consistent with that of COJO, while its cross-entropy loss function is retained instead of adopting the contrastive learning loss function. In addition, the compression ratio R is set within the range of [1/24, 1/2.5], and all training and testing are implemented over AWGN channels to simulate the image transmission and reconstruction process under different compression ratios. Meanwhile, the traditional encoding method (BPG + 3/4 LDPC + 64QAM) is also selected as a baseline for comparison.

Figure 7a presents the classification accuracy of COJO, SemCC [22], DeepSC [25], and the traditional method on the CIFAR-10 dataset at SNRs of 5 dB and 20 dB, while Figure 7b shows their PSNR values for image reconstruction on the same dataset. It should be noted that the COJO scheme in Figure 7 undergoes both semantic encoding compression and further compression via the SFS module, whereas SemCC [22] only undergoes a single semantic encoding compression step. As observed in Figure 7a, the proposed COJO scheme achieves higher classification accuracy than SemCC [22], DeepSC [25], and the traditional method at both 5 dB and 20 dB SNR. Furthermore, as the compression ratio R increases from 1/24 to 1/2.5, the classification accuracy of all models gradually improves. Meanwhile, the classification accuracy of all models at 20 dB SNR is higher than that at 5 dB SNR, which is consistent with expectations: a higher SNR results in less channel interference on image transmission, thereby improving model accuracy.

From Figure 7b, it can be observed that the image reconstruction quality of the proposed COJO scheme on the CIFAR-10 dataset is superior to that of SemCC [22] and DeepSC [25] at both 5 dB and 20 dB SNR, achieving higher PSNR values than the latter two methods. This indicates that the COJO scheme possesses stronger processing capability in image reconstruction tasks and can deliver higher image quality under different SNR conditions. Specifically, when the SNR decreases (i.e., at 5 dB), COJO exhibits better reconstruction performance than SemCC [22] and DeepSC [25], demonstrating stronger robustness against significant noise interference. At a higher SNR (20 dB), the PSNR of images reconstructed by COJO is still higher than that of SemCC [22], further highlighting its advantages under clearer image transmission conditions.

The above experimental results demonstrate that despite undergoing two stages of compression, the COJO scheme proposed in this work outperforms the comparison models in both image reconstruction and classification performance. This confirms that COJO can effectively preserve important image details even at low compression rates and low SNR, thereby exhibiting strong adaptability and robustness for joint image reconstruction and classification tasks in MA applications.

#### 3.2.4. The Complexity Analysis of COJO

The proposed COJO scheme takes into account the performance of both image reconstruction and classification tasks, and employs a semantic extraction module to further compress semantic features. To evaluate its complexity, this experiment adopted indicators such as floating-point operations (FLOPs) and model parameter count (Params) to quantitatively compare the COJO scheme with the other two comparison methods, as shown in Table 1. Compared with SemCC [22] and DeepSC [25], the COJO scheme can balance the performance of image reconstruction and classification, and can operate in more complex scenarios with superior overall performance.

It can be observed that the proposed algorithm not only increases the time and space complexity, but also raises the requirements for computational resources and power consumption during runtime. These increases in complexity are intended to support semantic communication systems in achieving more efficient information transmission, such as lower transmission rates and enhanced model robustness. In other words, the proposed COJO scheme trades off low complexity for low bit rates and robust transmission performance, thereby balancing the performance requirements of MA tasks.

#### 3.2.5. Subjective Comparison of Reconstruction Quality

**Figure 8:**
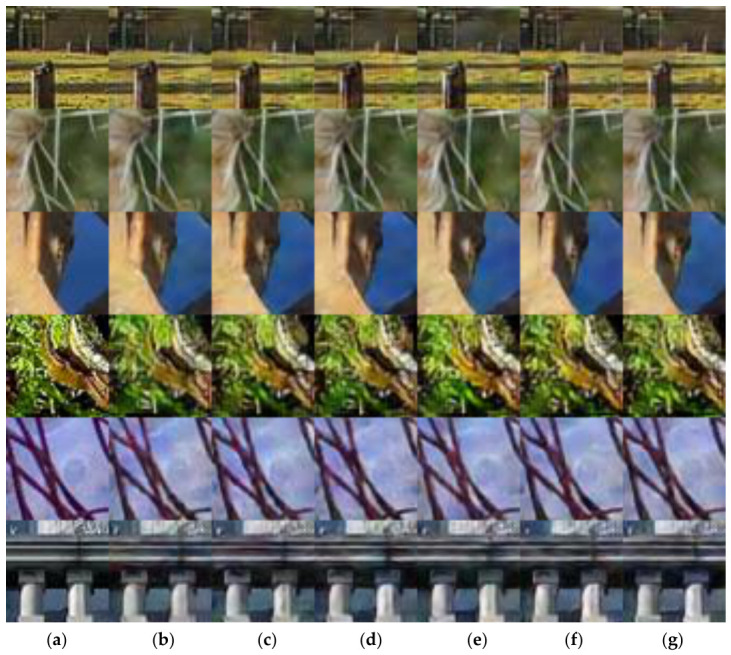
Observable results based on COJO in AWGN channel. (**a**) Original, (**b**) 0 dB, (**c**) 5 dB, (**d**) 10 dB, (**e**) 15 dB, (**f**) 20 dB, and (**g**) 25 dB.

To further analyze the image reconstruction quality, this experiment visualized the images reconstructed by the COJO scheme under different SNRs, specifically [0 dB, 5 dB, 10 dB, 15 dB, 20 dB, 25 dB]. Five test images were selected, and the compression ratio R was set to 1/6 for the experiment, aiming to compare the image reconstruction effect of the COJO scheme under different SNR conditions. Figure 8 presents the image reconstruction results under various additive white Gaussian noise (AWGN) channel conditions.

Specifically, when the SNR is 0 dB, the image reconstruction quality is already quite excellent. This indicates that the COJO algorithm can effectively resist the interference of channel noise and restore images with high visual quality even under low SNR conditions. As the SNR gradually improves, the quality of the reconstructed images further enhances, which further verifies the adaptability and stability of the COJO scheme under different channel condition levels.

Therefore, the proposed COJO scheme can effectively recover image information, resist the impact of channel noise, and demonstrate strong robustness—making it well-suited for MA semantic communication in complex application scenarios.

#### 3.2.6. Channel Adaptability

To further compare image reconstruction performance and present compact test curves, this experiment selects SemCC [22] as the baseline for comparison. The compression ratio R is set to 1/16, 1/12, 1/6 and 1/3, and the SNR varies from low to high to comprehensively evaluate the reconstruction robustness under diverse channel models. Evaluations are conducted on multiple datasets, including CIFAR-10 and STL-10. During training and testing, channel conditions and compression ratios are randomly sampled within the predefined ranges, which enables a full validation of reconstruction performance across various channel environments.

**Figure 9:**
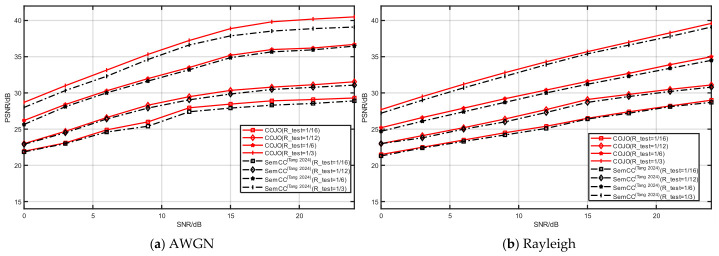
The reconstruction performance of CIFAR-10 under different channels [22].

**Figure 10:**
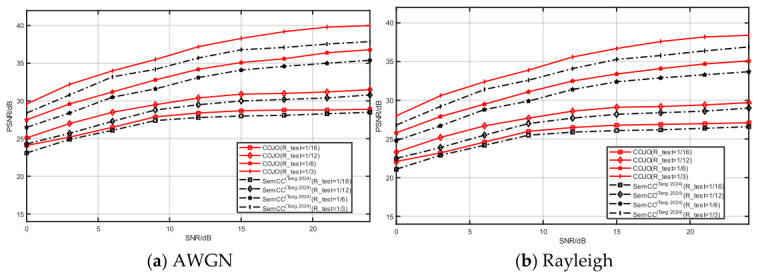
The reconstruction performance of STL-10 under different channels [22].

Figure 9 and Figure 10 illustrate the image reconstruction performance under AWGN and Rayleigh fading channels with different compression ratios. The proposed COJO achieves higher reconstruction PSNR than SemCC [22] at both low and high compression ratios. The results reveal that COJO delivers superior reconstruction quality and stronger channel robustness, highlighting its promising capability for image transmission in MA semantic communication systems.

#### 3.2.7. Generalization Experiment

To verify the generalization ability of the proposed COJO scheme, this experiment designates the CIFAR-10 and STL-10 datasets as training sets, and the CIFAR-100 and Kodak datasets as test sets, respectively. Training on the relatively simple CIFAR-10 and STL-10 datasets allows the model to learn basic image features. Testing on the CIFAR-100 and Kodak datasets, which are highly sensitive to complex tasks, can fully validate the model’s generalization ability when confronted with intricate scenarios. The training parameters of all the compared models remain consistent with those in the aforementioned experiments, with only the test sets replaced. To confirm the model’s adaptability to different channel environments, both the COJO scheme and the comparison model are tested on AWGN and Rayleigh fading channels, respectively.

**Figure 11:**
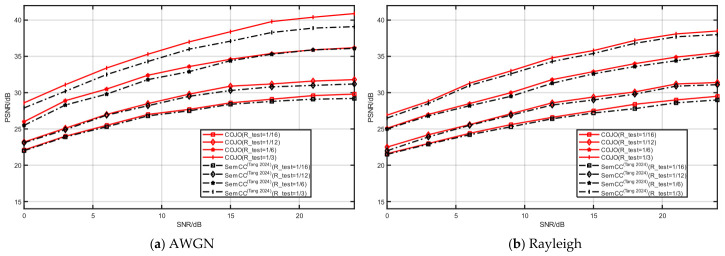
The reconstruction performance of CIFAR-100 under different channels [22].

**Figure 12:**
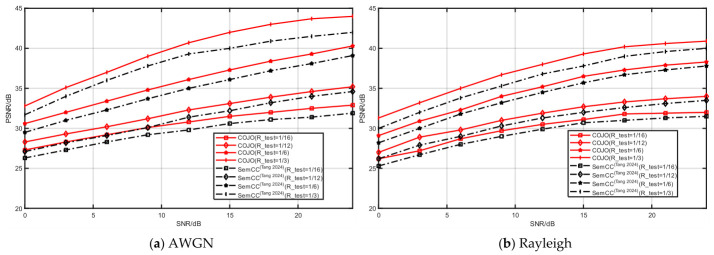
The reconstruction performance of Kodak under different channels [22].

Figure 11 and Figure 12 illustrate the reconstruction performance of the model on CIFAR-100 (trained on the CIFAR-10 dataset) and Kodak (trained on the STL-10 dataset). Typically, using training and test sets from different datasets may lead to a degradation in reconstruction performance. Fortunately, the test PSNR of the COJO scheme does not decrease significantly, with only a slight fluctuation. On both AWGN and Rayleigh fading channels, the reconstruction performance of the COJO scheme consistently outperforms that of SemCC [22]. Furthermore, it can be observed that, whether on the AWGN or Rayleigh fading channel, as the compression ratio R increases from 1/16 to 1/3, the COJO scheme exhibits superior data adaptability compared to SemCC [22], and the PSNR gap between the two schemes gradually widens. Particularly for larger image sizes, the performance advantage of the COJO scheme in image reconstruction becomes increasingly prominent.

Compared with the comparison methods, the COJO scheme more effectively extracts and preserves key features and information during image compression and restoration, ensuring excellent reconstruction performance and robustness when dealing with different datasets. These results demonstrate that even when the training and test datasets are distinct, the COJO scheme can still maintain relatively high reconstruction performance, possesses data reconstruction adaptability, and can be well-suited for more complex scenarios—making it applicable for MA systems.

## 4. Conclusions

This work proposes a COJO scheme based on contrastive learning for multi-task scenarios in multi-agent (MA) systems, aiming to alleviate the performance bottlenecks caused by limited transmission rates and varying channel conditions. To achieve a favorable performance trade-off between the two tasks, this work designs a TCM and a TRM for feature compression encoding, which substantially reduces the volume of transmitted data. By jointly optimizing the loss functions associated with semantic feature compression and image reconstruction, the proposed approach ensures improved classification accuracy while enhancing the quality of reconstructed images.

The contribution of the proposed COJO scheme to MA systems are elaborated on in detail as follows:

(1) It enables MA systems to accomplish tasks efficiently by leveraging both semantic information and refined features extracted via the TCM and TRM;
(2) It enhances the robustness of MA systems, even under unfavorable channel quality or constrained bandwidth conditions;
(3) It strengthens the dis-criminal capability for MA tasks, because the TCM considers the feature distributions corresponding to different tasks and effectively reduces the dimension of semantic information.

Accordingly, this work provides a valuable reference for the theoretical investigation and practical deployment of semantic communication technologies in MA-oriented applications.

## Figures and Tables

**Table 1:** The comparison of COJO’s complexity.

Models	FLOPs	Params	Memory Usage
SemCC [22]	0.24 G	5.79 M	144.20 MB
DeepSC [25]	0.26 G	5.40 M	146.20 MB
COJO (our)	0.29 G	6.80 M	147.78 MB

## Data Availability

The original contributions presented in this study are included in the article. Further inquiries can be directed to the corresponding author.
